# The relationship between atmospheric lead emissions and aggressive crime: an ecological study

**DOI:** 10.1186/s12940-016-0122-3

**Published:** 2016-02-16

**Authors:** Mark Patrick Taylor, Miriam K. Forbes, Brian Opeskin, Nick Parr, Bruce P. Lanphear

**Affiliations:** Department of Environmental Sciences, Faculty of Science and Engineering, Macquarie University Energy and Environmental Contaminants Research Centre, Sydney, NSW Australia; Centre for Emotional Health, Department of Psychology, Macquarie University, Sydney, NSW Australia; Macquarie Law School, Faculty of Arts, Macquarie University, Sydney, NSW Australia; Department of Marketing and Management, Faculty of Business and Economics, Macquarie University, Sydney, NSW Australia; Department of Health Sciences, Simon Fraser University, Vancouver, BC Canada

**Keywords:** Aggressive crime, Assault, Childhood, Lead exposure, Death

## Abstract

**Background:**

Many populations have been exposed to environmental lead from paint, petrol, and mining and smelting operations. Lead is toxic to humans and there is emerging evidence linking childhood exposure with later life antisocial behaviors, including delinquency and crime. This study tested the hypothesis that childhood lead exposure in select Australian populations is related to subsequent aggressive criminal behaviors.

**Methods:**

We conducted regression analyses at suburb, state and national levels using multiple analytic methods and data sources. At the suburb-level, we examined assault rates as a function of air lead concentrations 15–24 years earlier, reflecting the ubiquitous age-related peak in criminal activity. Mixed model analyses were conducted with and without socio-demographic covariates. The incidence of fraud was compared for discriminant validity. State and national analyses were conducted for convergent validity, utilizing deaths by assault as a function of petrol lead emissions.

**Results:**

Suburb-level mixed model analyses showed air lead concentrations accounted for 29.8 % of the variance in assault rates 21 years later, after adjusting for socio-demographic covariates. State level analyses produced comparable results. Lead petrol emissions in the two most populous states accounted for 34.6 and 32.6 % of the variance in death by assault rates 18 years later.

**Conclusions:**

The strong positive relationship between childhood lead exposure and subsequent rates of aggressive crime has important implications for public health globally. Measures need to be taken to ameliorate exposure to lead and other environmental contaminants with known neurodevelopmental consequences.

## Background

Environmental lead exposure is toxic to humans. Still, given the difficulty of proving that lead exposure causes harmful effects, and the cost of interventions, it has been difficult to implement primary prevention strategies to achieve lower levels of exposure. This is despite overwhelming evidence that there is no threshold or apparent safe level of lead exposure in its negative impact on intelligence, academic achievement and other neuro-cognitive and health outcomes [1–5]. The annual costs of childhood lead exposure are estimated to be up to $50 billion in the USA and €22.7 billion for France [6, 7]. However, the benefit of intervention to mitigate lead exposure is well established. It has been estimated that for each dollar spent to reduce lead exposure in housing, the benefit to society is $17 to $220 [8].

Australia is one the world’s largest producers and exporters of lead [9]. However, the majority of research on the neurocognitive and behavioral effects of lead exposure has been conducted in the USA and elsewhere. Despite emerging evidence from the USA that links early life lead exposure with antisocial behaviors, including conduct disorder, delinquency and crime [10–12], there is no published research on the effects of lead exposure on delinquency or criminality across subsets of Australian populations. In a multi-national study, Nevin [11] used estimates of Australia’s national blood lead trend to correlate to adulthood national criminal behaviours, identifying a strong association between preschool blood lead levels and subsequent crime rate trends. The prevailing approach to understanding causes of adult crime focuses heavily on factors such as parenting style, socioeconomic status, and peer groups [13]. The paucity of research examining the links between lead exposure and criminality is surprising given the strong evidence that childhood lead exposure is linked to a variety of socio-behavioral problems that are precursors for criminal behavior [12, 14–17].

Historically, lead exposure in Australia has been dominated by three sources: (i) lead paint, (ii) leaded petrol and (iii) mining and smelting emissions, all of which pose a potential risk to human health. Blood lead levels in the Australian population have fallen since the final removal of lead from petrol in 2002 [18, 19] together with the reduction of allowable lead in paint to 0.1 % in 1997. However, the legacy of leaded petrol emissions and the renovation of premises that once used lead paint continue to pose potential environmental hazards, particularly in the older parts of Australian cities [20]. Kristensen [18] calculated that emissions from seven decades of leaded petrol use (1932–2002) exceeded 240,000 tonnes, dwarfing lead mining and smelting sources [21]; there is a strong relationship between these emissions and contemporaneous childhood blood lead levels (*r* = 0.970, *p* < 0.00001) [18]. Mining and smelting operations have also been a major source of lead emissions in Australia [22, 23]. Examples of historical exposure include Port Kembla and Boolaroo in the state of New South Wales (NSW), which are considered in this study; while examples of ongoing exposure include Broken Hill (NSW), Mount Isa (Queensland) and Port Pirie (South Australia) for which relevant data were not available. At Port Kembla and Boolaroo, children’s mean blood lead levels were elevated during smelting operations - more than three times the current Australian intervention level of 5 μg/dL [24, 25].

This study addresses the research gap by examining the relationship between lead exposure of select Australian populations (including children, who are the most vulnerable section of the population to lead toxicity) and subsequent criminality during adolescence and early adulthood. We test the hypothesis that there is a significant correlation between shifts in lead exposure and rates of aggressive crime in later life, and we do this at suburb, state and national levels using multiple methods.

## Methods

We operationalize the hypothesis as follows. For the suburb-level analysis, we examine rates of assault (an impulsive and aggressive crime) over time as a function of air lead concentrations 15–24 years earlier in NSW suburbs where sufficient data are available. As a test for discriminant validity, we also examine the relationship between air lead concentrations and fraud rates in the same suburbs; fraud being a non-impulsive and non-aggressive crime. We supplement our analysis by examining the relationship between lead exposure and later aggressive crime at different geographic scales by investigating state and national data over time. Due to restrictions on data availability, we utilize total lead emissions from the combustion of leaded petrol as a proxy for lead exposure, and deaths by assault as a proxy for aggressive crime.

### Study sites

We conducted suburban analyses of air lead concentrations and criminal behaviors in NSW. Suburbs were included if air lead data were available for at least 30 years. The six suburbs were: Boolaroo, Earlwood, Lane Cove, Port Kembla, Rozelle and Rydalmere. Table 1 summarises the descriptive statistics for the six sites. The average population at risk of exposure in these suburbs over the relevant census period (1976–1991) ranged from 1392 in Boolaroo to 17,729 in Earlwood. The Sydney central business district (CBD) also had these data available, but it was excluded due to the transience of the resident population and the likelihood that local residents were not responsible for the exceptionally large number of recorded assaults. The average annual assault rate in Sydney CBD from 1995 to 2014 was 10,730 per 100,000 population; the next highest was Port Kembla with 1627 per 100,000 population. The suburbs included in the study varied in size, socio-demographic characteristics, and air lead concentrations (see Table 1). Four of the six are metropolitan locations, which were impacted primarily by leaded petrol emissions, while Boolaroo and Port Kembla are regional communities with a history of lead, zinc and copper smelting that caused significant environmental lead pollution. We also examined aggregated death by assault data from each Australian state and territory, as well as national data. The average population at risk of exposure over the relevant period (1958–2002) ranged from 5.39 million in NSW to 119,370 in the Northern Territory [26].

### Data sources

All available air lead data were extracted from NSW Environment Protection Authority records for the suburb-level analyses. The values were reported as micrograms per cubic metre (μg/m^3^) from air monitoring stations, dating as far back as 1973. The annual air lead value for each site was calculated as the mean of all readings for each year. Where there was more than one monitoring station in a suburb, the station with the most complete data was used to maximize reliability in the variation in lead levels over time.

Annual atmospheric lead emissions (tonnes per annum) by state were taken from Kristensen [18] for the state-level analyses. These data were derived from the volume of leaded petrol sales, the known but varying concentrations of lead in petrol over time, and the percentage of lead emitted from combustion. The state-level lead data were aggregated for the purpose of the national-level analysis. Because petrol lead emission data are less specific in terms of exposure compared to suburb-level data based on direct air monitoring, it was anticipated that resulting state and national analyses would be less precise.

Crime data for the suburb-level analyses, were extracted from the Computerised Operational Policing System (COPS) of the NSW Police Force in February 2015. The records of assaults reported to police were provided by the NSW Bureau of Crime Statistics and Research, and included statistics from 1995 to 2014. Rates of assault were used to operationalize impulsive aggression-related crimes. The assault statistics included domestic and non-domestic violence, and assaults on police. Rates of fraud were used as a control for non-impulsive and non-aggressive crime. Total assault rates and fraud rates per 100,000 population were calculated for the postcode (zipcode) corresponding to each of the six suburbs. Customized population data were sourced from the Australian Bureau of Statistics (ABS) based on official five yearly census data.

Customized data on deaths by assault were obtained from the Australian Institute of Health and Welfare’s General Record of Incidence of Mortality books for the state-level analyses. The relevant deaths comprised those in categories X85–Y09 of the latest International Classification of Disease (ICD–10), and equivalent categories in prior iterations of ICD–10 [27]. These categories include homicides and injuries inflicted by another person with intent to injure or kill, by any means. A breakdown of deaths by state was available only for the period 1964–2012. The number of deaths per state was then scaled by the mid-year resident population of that state using demographic data from the ABS to determine the deaths by assault per 100,000 population.

The crime data reveal marked differences in rates of offending by age. This phenomenon has long been recognized in criminological literature across time, social contexts, demographic groups and crime types, although its causes are contested [28]. The peak age in Australia for recorded crime comprising acts intended to cause injury (including assaults) is 15–24 years [29]. A somewhat similar age peak occurs in relation to crimes of fraud or deception, although it is far less pronounced. The ‘age-crime curve’ is relevant to determining the optimal time lag between childhood lead exposure and later criminality when investigating correlations.

### Data analysis

All suburb-level analyses were controlled for major socio-demographic correlates of crime, including: the proportion of the population aged 15–24; the proportion of the population who completed secondary school; and the median household income per annum. These data were extracted by the ABS from the 5 yearly Census of Population and Housing (conducted in 1991, 1996, 2001, 2006 and 2011) for each suburb based on place of usual residence. We used the census data that was most contemporary to the annual crime data. Median household income was adjusted for inflation (i.e., analysed in 2014 Australian dollars) using the Reserve Bank of Australia’s inflation calculator [30].

All available data were used for each of the six suburbs, and missing observations were treated as missing at random. Preliminary analyses were run to examine the direct relationships between lead in air concentrations and crime rates at each year on the 15–24 year age-crime curve. A random intercept linear mixed-effects model was run in SPSS version 22 using maximum likelihood estimation, and the relationships between observations within each suburb were accounted for using a random subject factor. This model was used because the assumptions of regression were not appropriate (e.g., observations were not independent). Omega-squared (ω^2^) values were calculated to provide an approximation of the variance accounted for by each variable, i.e., pseudo-R^2^ [31]. Covariates were subsequently included in the best mixed model to examine the predictive validity of lead exposure after controlling for major correlates of crime. To test for discriminant validity with non-impulsive crime, models were tested using fraud rates as the dependent variable.

For the state-level analyses, death rates (deaths by assault per 100,000 population) were plotted against lead petrol emissions (tonnes/year) for each state, with 10 different time lags (15–24 years), and linear regression lines were fitted and coefficients of determination calculated. Lead petrol emissions for the Australian Capital Territory were not available separately as they are included in the NSW data [18]. Corresponding death data were aggregated accordingly. The number of data points varied according to the time lag applied because the available emission data (1958–2002) and death data (1964–2012) were not congruent.

## Results

**Fig. 1:**
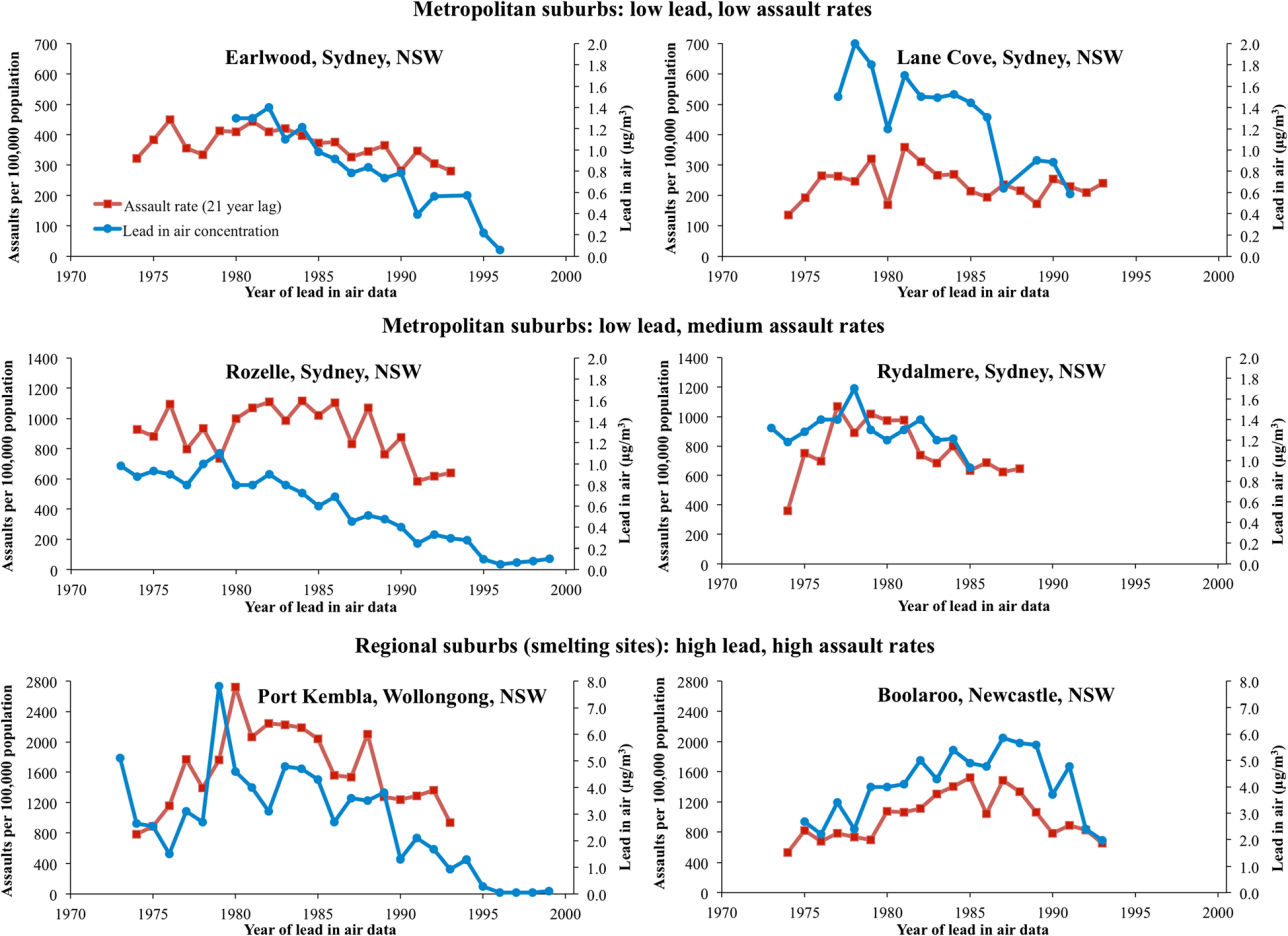
Lead in air concentrations and assault rates for six suburbs, 1973–1999

**Fig. 2:**
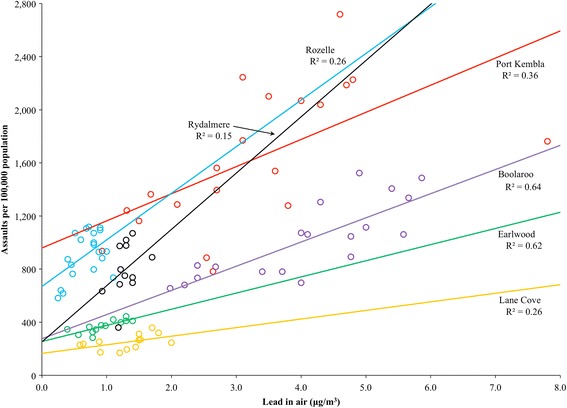
Scatterplot showing the relationships between lead in air concentrations and assault rates 21 years later for all six suburbs

At the suburb level, the zero-order correlations between lead in air and assault rates peaked at a 21-year lag for most sites. The correlations at the 21-year lag were strong and significant for all sites (range *r* = .506 to *r* = .802, all *p* values ≤ .022) except Rydalmere (*r* = .386, *p* = .215), which had the shortest time series (see Figs. 1 and 2). Without adjusting for major socio-demographic correlates of crime, lead in air accounted for 26–64 % of the variance (ω^2^) in assault rates at each site 21 years later (15 % for Rydalmere). It is notable that in the four metropolitan suburbs, the data points are tightly clustered, with mean annual lead in air levels markedly lower than in the two smelting communities of Boolaroo and Port Kembla (Fig. 2). The maximum annual value was 5.9 μg/m^3^ (1987) in Boolaroo and 7.8 μg/m^3^ (1979) in Port Kembla. This can be compared to the current national air lead standard of 0.5 μg/m^3^ (expressed as an annual average) [32]. Lead in air concentrations in metropolitan suburbs also exceeded 0.5 μg/m^3^ until some years after the introduction of unleaded petrol in 1985 [18].

Direct effects between air lead and assault rates across all suburbs were examined using linear mixed-effects models for time lags between 15 and 24 years. The relationship peaked in the middle of the age-crime curve, with the strongest direct effect for lead in air as a predictor of assault rates at the 21-year lag (see Table 2). In this mixed model, every additional μg/m^3^ of lead in air was associated with an increase of 196 assaults per 100,000 population, and lead in air accounted for 38.4 % of the variance in assault rates.

**Table 1:** Summary statistics for the six suburb sites

Suburb (number of years with complete lead and crime data at 21-year lag)	Suburb data in the years with lead data		Suburb information in the years with crime data (mean ± std devn)					
	Years with lead data	Air lead μg/m^3^	Years with crime data	Assault rates per 100,000	Fraud rates per 100,000	Population aged 15–24 (%)	Median weekly income	Population finished secondary school (%)
Boolaroo (*n* = 19)	1975–1993	4.06 ± 1.254	1995–2014	990.33 ± 297.95	219.48 ± 120.993	11.69 ± 0.498	965.61 ± 90.555	27.57 ± 3.418
Earlwood (*n* = 13)	1980–1996	0.82 ± .394	1995–2014	367.15 ± 49.696	240.07 ± 81.948	12.00 ± 1.602	1359.34 ± 106.04	50.41 ± 5.126
Lane Cove (*n* = 14)	1977–1991	1.32 ± .426	1995–2014	238.35 ± 54.049	449.89 ± 280.851	12.43 ± 1.273	1985.33 ± 275.179	72.15 ± 4.917
Port Kembla (*n* = 20)	1974–1999	2.68 ± 1.906	1995–2014	1627.11 ± 530.602	365.50 ± 140.128	12.30 ± .963	779.45 ± 78.714	28.89 ± 4.539
Rozelle (*n* = 20)	1973–1999	.57 ± .334	1995–2014	908.35 ± 173.472	790.67 ± 240.948	8.70 ± 1.854	2321.95 ± 470.56	72.38 ± 7.961
Rydalmere (*n* = 12)	1973–1985	1.29 ± .175	1995–2014	769.87 ± 188.164	487.81 ± 228.232	11.96 ± 1.00	1296.60 ± 89.252	49.00 ± 5.960
All sites (*n* = 98)	–	1.84 ± 1.645	–	818.90 ± 537.018	422.86 ± 272.331	11.49 ± 1.819	1458.11 ± 602.461	50.11 ± 19.216

**Table 2:** Mixed model analyses of the direct effects between air lead and assault rates for all six suburbs with time lags between 15 and 24 years

Time Lag (number of cases with complete information)	F	df	*p*	Fixed effects (SE)	ω^2^ (%)
15 years (*n* = 87)	.857	86.940	.357	30.50 (32.951)	0.46
16 years (*n* = 90)	.045	89.434	.832	6.20 (29.191)	−0.09
17 years (*n* = 93)	6.534	92.673	.012	72.23 (28.256)	5.54
18 years (*n* = 96)	14.021	95.874	.000	104.87 (28.007)	11.66
19 years (*n* = 97)	29.922	96.784	.000	145.61 (26.619)	22.88
20 years (*n* = 98)	34.989	97.895	.000	159.66 (26.993)	25.60
21 years (*n* = 98)	61.285	97.761	.000	196.05 (25.044)	38.38
22 years (*n* = 98)	41.507	97.864	.000	180.09 (27.954)	28.70
23 years (*n* = 94)	7.064	93.865	.009	85.75 (32.264)	5.13
24 years (*n* = 89)	9.613	88.995	.003	99.22 (32.003)	7.72

*F* F-test, *df* degrees of freedom, *p p*-value, *Fixed effects* the estimated change in assaults per 100,000 population for a 1 μg/m^3^ increase in lead in air, *SE* standard error, *ω* ^*2*^ an estimate of the amount of variance accounted for by lead in air

Major socio-demographic correlates of crime were subsequently added as covariates in the 21-year lag mixed model. Primary analyses included socio-demographic covariates for the years in which the assaults were committed. As suggested by Bellinger [14] we also examined models that controlled for socio-demographic variables at the time of lead exposure, but these variables did not reach significance in either model and consequently were excluded from the analyses to avoid multicollinearity between the two sets of socio-demographic variables.

Accounting for socio-demographic covariates, lead in air remained a strong predictor of assault rates. For every additional μg/m^3^ of lead in air, assault rates 21 years later increased by 163 per 100,000 population (see Table 3). Lead in air was the strongest predictor in the model, accounting for 29.8 % of the variance in assault rates 21 years later. By comparison, the proportion of the population aged 15–24 accounted for 5.4 % of the variance, and the proportion of the population who completed secondary school accounted for 5.0 %. Median income was not a significant predictor in the model. The proportion of people aged 15–24 had the reverse effect on assault rates to that anticipated (i.e., each additional percentage of the population aged 15–24 was related to a decrease in assaults). This is most likely related to the restricted variance in these variables when expressed as a proportion, and the overlap between the three socio-demographic variables.

**Table 3:** Parameter estimates in the full mixed model (*n* = 98). Dependent variable: assault rates per 100,000 population

	F	df	*p*	Fixed effects (SE)	ω^2^ (%)
Lead in air (μg/m^3^)	39.064	95.375	<.0005	162.94 (26.070)	29.78
Proportion of the population aged 15–24 years	5.509	93.434	.021	−85.99 (36.636)	5.41
Proportion of the population who completed secondary school	4.128	91.577	.045	−16.58 (8.159)	4.96
Median income (2014 Australian dollars)	.003	95.430	.958	.01 (.251)	0.0

*F* F-test, *df* degrees of freedom, *p p*-value, *Fixed effects* the estimated change in assaults per 100,000 population for a 1 unit increase in the independent variable, *SE* standard error, *ω* ^*2*^ an estimate of the amount of variance accounted for by the independent variable

As a test for discriminant validity, mixed models that examined the relationship between lead in air and fraud rates were also examined for the 15–24 age-crime curve. There were some small statistically significant relationships, but the largest effect of lead as a predictor of fraud rates (lagged 15 years) accounted for only 5.5 % of the variance. It is apparent that the explanatory power of lead in air is minimal in relation to fraud rates, which contrasts markedly with assault rates.

**Fig. 3:**
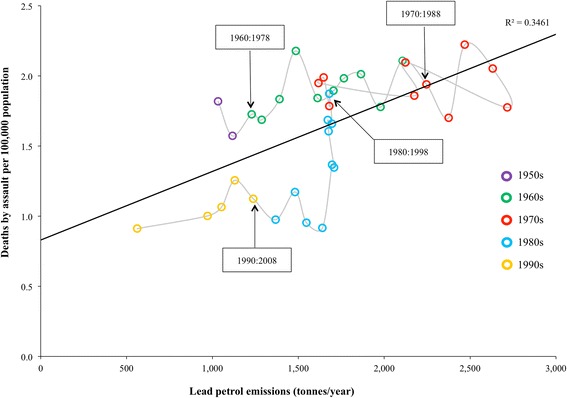
Scatterplot showing the relationship between lead petrol emissions and death by assault rates 18 years later for NSW

At the state level, strong positive correlations between petrol lead emissions and death by assault rates were found only for the states with the largest populations, highest population densities and greatest petrol lead emissions, namely, NSW and Victoria. In these states, correlations peaked at the 18-year lag, which reflects the age-crime curve described in the literature [28]. A simple linear regression model showed that lead emissions in NSW accounted for 34.6 % of the variance in death by assault rates 18 years later. Every 2000 additional tonnes of lead emitted was associated with one additional death. Moreover, there is a clear temporal pattern to the data. The death by assault rate increases over the period 1976 to 1992, corresponding to increases in petrol lead emissions 18 years prior. In the subsequent period from 1992 to 2012 the death by assault rate falls, reflecting the reduction in petrol lead emissions 18 years prior. This hysteresis effect is shown in Fig. 3. In Victoria, the most densely populated state, a simple linear regression model showed that lead emissions accounted for 32.6 % of the variance in death by assault rates 18 years later. Every 1667 additional tonnes of lead emissions was associated with one additional death. The hysteresis pattern observed in the NSW data was also evident in the Victorian data. In states and territories with low population densities and low absolute emission levels, the correlation was negative.

At a national level, the data also demonstrated a positive correlation between lead emissions and death by assault rates, but the association was weak. National lead emissions accounted for only 7 % of the variance in national death by assault rates 18 years later, as the health and behavioral effects of lead emissions are dissipated at larger geographic scales.

## Discussion

Our study tested the hypothesis that there is a significant correlation between air lead exposure and rates of aggressive crime in later life. The results demonstrate that after controlling for major socio-demographic correlates of crime there is a strong positive relationship between lead in air levels and subsequent crime rates. This has important implications for public health globally.

This is the first Australian study to test the hypothesis that lead exposure is associated with subsequent aggressive criminal behaviors at a range of spatial scales. Lead in air concentrations accounted for 29.8 % of the variance in assault rates 21 years later in the six localities measured, after adjusting for socio-demographic covariates. In the most populous Australian states of NSW and Victoria, total lead petrol emissions accounted for 34.6 and 32.6 %, respectively, of the variance in death by assault rates 18 years later. Given the variety of possible determinants of criminal behavior, these are remarkable findings. The R^2^ values for the states are not atemporal, but reflect secular trends in the variables as indicated by the hysteresis loop in Fig. 3.

These results are robust because the study relies on statistics from official government and industry agencies that have collected relevant datasets independently of each other. We operationalized our hypotheses using two variables for lead exposure (lead in air concentrations and annual lead petrol emissions) and three variables for recorded crime (assault, death by assault and fraud) across different spatial and temporal scales. The suburbs varied in size, lead levels, crime rates, and socio-demographic characteristics, and a variety of statistical methods were utilized to analyze the data. Consequently, the consistency of the relationships across the models suggests the results are robust.

The association between lead in air and lagged assault rates at the suburb scale exists regardless of whether the source of lead is smelting or petrol. Five of the six sites have positive and significant correlations, with the sixth (Rydalmere) being affected by the small sample size (Fig. 1). This is important because the temporal pattern of lead emissions varies across sources and sites and yet the outputs remain compatible with our hypothesis. Notably, the strongest relationship was found in the smelting town of Boolaroo (R^2^ = 0.64), and the third highest was in the smelting town of Port Kembla (R^2^ = 0.36); these suburbs had the highest levels of lead pollution. Removal of a single outlier in the lead in air data set for Port Kembla (7.8 μg/m^3^, 1979) lifted R^2^ to 0.59.

The study suggests that features of the physical environment, in this case atmospheric pollution, may be more important than previously considered in explaining early adult criminality. After adjusting for major socio-demographic variables (population age distribution, education, income), lead in air remained the largest determinant of variance in assault rates. It accounted for 5.5 times as much of the variance as the single most important socio-demographic factor and 2.8 times as much as the combined socio-demographic covariates (Table 3).

The study outcomes are consistent with the neuro-psychological literature, which suggests that the principal behavioral traits affected by childhood lead exposure are reduced impulse control and related impacts on aggressive behaviors [11, 12, 33–36]. Childhood blood lead exposure is also associated with reduced adult brain volume in the prefrontal and anterior cingulate cortex areas that are responsible for executive functioning, mood regulation and decision-making [37].

Our study reveals the importance of lead in air as a determinant of rates of aggressive crime. This is consistent with Marcus et al.’s [10] meta-analysis of >8000 children and adolescents, which showed a significant association between lead exposure and conduct problems in later life. By contrast, fraud, which is a non-impulsive, non-aggressive crime, was only associated weakly with prior exposure to lead in air (ω^2^ ≤ 5.5 %).

This study has data limitations that are typical of other ecological studies, like herd immunity. The measured correlations between lead in air and subsequent rates of aggressive crime may be underestimated due to lack of congruence between the populations exposed to lead and the populations measured for later criminal behaviors [38]. This is a consequence of the deaths and out-migration of some lead-exposed individuals, the births over the period subsequent to the measurement of exposure to lead, and the in-migration of other individuals who have been exposed to lead at unknown concentrations and localities. Quantifying the impact of these processes is difficult due to limited data availability at the suburb level. Over the period 2001–2014, which is only part of the study time period, there was population growth in all six suburbs: Earlwood 3.2 %, Port Kembla 3.9 %, Boolaroo 4.4 %, Lane Cove 10.9 %, Rydalmere 16.6 % and Rozelle 30.7 %. All but the last suburb were below the national average growth of 21.9 % for that period [39]. There was also substantial turnover in the membership of the populations of all six suburbs due to migration. The percentages of people aged over 5 years who lived in a different local area 5 years before the 2011 census were substantial: Earlwood 22.0 %, Port Kembla 23.3 %, Boolaroo 26.9 %, Rydalmere 28.0 %, Lane Cove 36.8 % and Rozelle 49.4 %. Whilst more of the in-movers to the high turnover suburbs of Lane Cove and Rozelle came from other parts of Australia, there were also significant numbers who moved from overseas. Of the population aged 5 and over in 2011, 11.9 % of Rozelle’s population and 9.9 % of Lane Cove’s population were living outside Australia 5 years earlier [39].

With respect to lead in air, it would be desirable to have broader and more detailed spatial and temporal coverage. However, we have used the best available data for which there are also corresponding crime data. For the suburb level analysis, lead in air concentrations were sourced from a single air monitoring station to characterize exposure across the selected geographic area. For the state and national analyses, lead petrol emissions were estimated from petrol sales and are a proxy for population lead exposure. With respect to crime rates, data on assaults are those reported to police, which may be under-inclusive due to unreported crime or over-inclusive due to unsubstantiated allegations. Assault data is based on the suburb where the assault took place, not the offender’s residence, which might be more closely linked with lead exposure. Similarly, death by assault data are based on the state or territory in which the death was registered, not the residence of the person who caused the death. Nonetheless, we have found noteworthy results in the face of limitations that might have been expected to obscure the relevant relationships.

Finally, the study suggests productive areas for future research with respect to lead and other neurotoxic metals [40]. This study is one of association not causation. More specificity could be obtained by examining the blood lead concentrations of individuals and undertaking a prospective longitudinal study of their behavioral responses. While a few studies have achieved this benchmark [10, 12, 34], more research is required across different populations and contaminants. Better data will help formulate evidence-based policies to improve health and social outcomes.

Taken together, the results of the present study highlight that atmospheric lead standards require systematic review by national and international agencies. At present, standards vary widely. For example, the lead in air standard is 0.5 μg/m^3^ (annual) (1 μg/m^3^, seasonal) in China, 0.5 μg/m^3^ in Australia and 0.15 μg/m^3^ in the USA. The method for calculating acceptable levels also varies. In Australia the standard is based on an annual average, with no upper limit on short-term spikes; in the USA it is based on a 3-month rolling average, which is more restrictive on polluters. Future revisions of lead in air standards need to be tied to demonstrable health outcomes, cognizant of their impact on anti-social behaviors.

Measures need to be taken to reduce or eliminate extant sources of atmospheric lead pollution wherever practicable. Exposures from these sources have the potential to increase anti-social behaviors and impose unnecessary societal costs. These sources include existing mining and smelting operations in Australia and elsewhere, and lead petrol consumption in countries where it is still sold: Algeria, Iraq, and Yemen [41]. In these countries, some 103 million people remain at risk from the use of lead petrol [42]. There are also policy implications for communities that have been historically affected by the deposition of atmospheric lead in populated places such as homes, gardens, playgrounds and schools. These depositions present an ongoing risk because the half-life of environmental lead exceeds 700 years [43].

## Conclusions

This study found a robust relationship between lead in air and subsequent rates of aggressive crime at suburb, state and national population levels using multiple analytic methods and data sources. These results add to the existing body of literature that highlights the sequelae of lead exposure. Fortunately, exposure to lead is preventable and remedial intervention is cost effective [8]. Given the overwhelming evidence that there is no safe lower threshold for lead toxicity, remediation programs are essential to mitigate these effects and should be a clear priority for immediate policy change.

## Abbreviations

ω^2^: Omega-squared
ABS: Australia Bureau of Statistics
ICD-10: International Classification of Diseases 10^th^ Revision
SPSS: Statistical Package for the Social Science
X85-Y09: classified under ICD-10 as external causes of morbidity and mortality and are inclusive of homicides and injuries inflicted by another person with the intent to injure or kill, by any means.
μg/dL: micrograms per decilitre
μg/m^3^: micrograms per cubic metre
