# Erratum to: The relationship between atmospheric lead emissions and aggressive crime: an ecological study

**DOI:** 10.1186/s12940-016-0204-2

**Published:** 2016-12-30

**Authors:** Mark Patrick Taylor, Miriam K. Forbes, Brian Opeskin, Nick Parr, Bruce P. Lanphear

**Affiliations:** 1 Department of Environmental Sciences, Faculty of Science and Engineering, Macquarie University Energy and Environmental Contaminants Research Centre, Sydney, NSW Australia; 2 Centre for Emotional Health, Department of Psychology, Macquarie University, Sydney, NSW Australia; 3 Macquarie Law School, Faculty of Arts, Macquarie University, Sydney, NSW Australia; 4 Department of Marketing and Management, Faculty of Business and Economics, Macquarie University, Sydney, NSW Australia; 5 Department of Health Sciences, Simon Fraser University, Vancouver, BC Canada

## Erratum

Subsequent to the publication of our article [1], we have now made the data available on figshare with the doi https://dx.doi.org/10.6084/m9.figshare.4109784.v1.
